# A mouse infection model and long-term lymphatic endothelium co-culture system to evaluate drugs against adult *Brugia malayi*

**DOI:** 10.1371/journal.pntd.0010474

**Published:** 2022-06-07

**Authors:** Amy E. Marriott, Julio Furlong Silva, Nicolas Pionnier, Hanna Sjoberg, John Archer, Andrew Steven, Dale Kempf, Mark J. Taylor, Joseph D. Turner

**Affiliations:** 1 Centre for Drugs and Diagnostics & Centre for Neglected Tropical Diseases, Department of Tropical Disease Biology, Liverpool School of Tropical Medicine, Pembroke Place, Liverpool, United Kingdom; 2 Pharmaceutical R&D, AbbVie, North Chicago, Illinois, United States of America; University of Liverpool, UNITED KINGDOM

## Abstract

The development of new drugs targeting adult-stage lymphatic filarial nematodes is hindered by the lack of a robust long-term *in vitro* culture model. Testing potential direct-acting and anti-*Wolbachia* therapeutic candidates against adult lymphatic filariae *in vitro* requires their propagation via chronic infection of gerbils. We evaluated *Brugia malayi* parasite burden data from male Mongolian gerbils compared with two immune-deficient mouse strains highly susceptible to *B*. *malayi*: CB.17 Severe-Combined Immmuno-Deficient (SCID) and interleukin-4 receptor alpha, interleukin-5 double knockout (IL-4Rα^-/-^IL-5^-/-^) mice. Adult worms generated in IL-4Rα^-/-^IL-5^-/-^ mice were tested with different feeder cells (human embryonic kidney cells, human adult dermal lymphatic endothelial cells and human THP-1 monocyte differentiated macrophages) and comparative cell-free conditions to optimise and validate a long-term *in vitro* culture system. Cultured parasites were compared against those isolated from mice using motility scoring, metabolic viability assay (MTT), *ex vivo* microfilariae release assay and *Wolbachia* content by qPCR. A selected culture system was validated as a drug screen using reference anti-*Wolbachia* (doxycycline, ABBV-4083 / flubentylosin) or direct-acting compounds (flubendazole, suramin). BALB/c IL-4Rα^-/-^IL-5^-/-^ or CB.17 SCID mice were superior to Mongolian gerbils in generating adult worms and supporting *in vivo* persistence for periods of up to 52 weeks. Adult females retrieved from BALB/c IL-4Rα^-/-^IL-5^-/-^ mice could be cultured for up to 21 days in the presence of a lymphatic endothelial cell co-culture system with comparable motility, metabolic activity and *Wolbachia* titres to those maintained *in vivo*. Drug studies confirmed significant *Wolbachia* depletions or direct macrofilaricidal activities could be discerned when female *B*. *malayi* were cultured for 14 days. We therefore demonstrate a novel methodology to generate adult *B*. *malayi in vivo* and accurately evaluate drug efficacy *ex vivo* which may be adopted for drug screening with the dual benefit of reducing overall animal use and improving anti-filarial drug development.

## Author summary

Lymphatic filariasis (LF), the cause of elephantiasis, and onchocerciasis, the cause of river blindness, are Neglected Tropical Diseases (NTDs) prioritised for elimination as a public health problem. New drugs which cure filarial infection are required to accelerate elimination of these diseases. There is a lack of drug screening systems to accurately assess treatment efficacy of new candidate drugs, particularly for LF. Here we describe new methods to grow large yields of the causative agent of lymphatic filariasis, *Brugia malayi*, in selective immune-deficient mice and maintain female parasites outside of the body, in combination with a bi-layer of lymphatic endothelial cells, for periods of up to 3 weeks without loss of viability. We demonstrate the application of this system to test the efficacy of reference and experimental drug compounds including drugs targeting the filarial symbiotic bacterium, *Wolbachia*. The novel production and maintenance of female *B*. *malayi* described here will have useful future applications to more accurately determine candidates for onward clinical testing as new filariasis cures whilst reducing animal use, costs, and drug development time-frames.

## Introduction

Lymphatic filariaisis (LF), caused by the vector-borne parasitic nematodes *Wuchereria bancrofti*, *Brugia malayi* and *Brugia timori*, presents a significant global health burden with an estimated 51 million people currently infected in tropical regions of the world [1].

Clinically, LF manifests as elephantiasis and hydrocele in approximately 40 million individuals, making it a leading cause of global disability [2]. Lymphatic filariasis is prioritised for elimination as a public health problem by 2030. The current elimination strategy relies on annual mass drug administration (MDA) of albendazole alone, or in combination with either or both ivermectin and diethylcarbamazine, which target the transmissible stage of the disease, the microfilariae (mf). In Africa, in regions of overlapping onchocerciasis and/or loiasis endemicity, delivery of albendazole- and ivermectin-based MDA drug regimens for up to 6 years with high population coverage is required to block transmission of infection. Currently, MDA is still needed in 49 countries, with 13 countries having not fully scaled up their elimination programmes, and five countries yet to commence MDA [3].

Development of a safe and effective short-course curative (macrofilaricidal) drug or, alternatively, a drug which permanently sterilises female adult lymphatic filariae, would be an important new tool to dock the long-tail of filariasis elimination, particularly in transmission ‘hot-spots’ and hard-to-reach areas currently without MDA delivery, or where existing strategies are failing [4].

Novel macrofilaricidal drug candidates, targeting nematode molecules or the intracellular filarial symbiont, *Wolbachia*, are identified by screening chemical compound libraries using a variety of *in vitro* assays. Screening drug activity against adult stage lymphatic filariae in particular is hampered due to low throughput and short life-span *in vitro* which jeopardises accurate evaluations of drug efficacy in culture due to decline in viability. To increase throughput and extend culture periods, anti-filarial or anti-*Wolbachia in vitro* drug screens which utilise more accessible and/or abundant life cycle stages; namely the mf [5, 6] or infectious L3 stage [7] and other, surrogate, non-LF filarial parasites such as *Litomosoides sigmodontis* [8], *L*. *loa* [9], *Mansonella* perstans [10] or *Onchocerca spp*.[11, 12] have been validated and implemented. Whilst useful for initial screening, a caveat to these systems is that anti-filarial or anti-*Wolbachia* activities may not translate to the target adult stages of lymphatic filariae due to variation in stage- or species-specific expression of nematode drug targets or relative yields and cell division rates of *Wolbachia* symbionts.

A further impediment to macrofilaricidal drug discovery is that there is no established system of maintaining filarial life cycles outside of an animal host. Whilst there is no suitable permissive rodent model of *Wuchereria bancrofti* or *Onchocerca volvulus*, the Mongolian Gerbil, *Meriones unguiculatus*, is the standard laboratory model of *Brugia malayi* infection, and the closely related animal filariae, *Brugia pahangi* [13, 14]. Since the 1970s, Gerbils have been utilised to provide all life cycle stages for *Brugia spp*. for drug screening *in vitro* as well as onward therapeutic evaluations *in vivo*. Productivity in terms of numbers of adults and mf produced is highly variable, declines with age of infection and is generally low yielding, especially for *B*. *malayi*. More recently, Severe-Combined ImmunoDeficient (SCID) mice, deficient in adaptive immunity, have been appraised as long-term fully susceptible hosts for *B*. *malayi* and the human filarial pathogen, *Loa loa*, and have been subsequently validated as drug screening models [15–17]. *Brugia malayi* infection success and variability in adult yields are more consistent in SCID mice compared with gerbils [18]. We have recently identified that adaptive immune control of *B*. *malayi* in mice is dependent on interleukin (IL)-4 receptor-alpha (Rα) macrophage activation mediating recruitment of a vigorous tissue eosinophilia [19]. By ablating gene function of IL-4Rα and the eosinophil growth factor, IL-5, full development of *B*. *malayi* in mice is also possible [20].

Here, we compare two immuno-deficient mouse strains; CB.17 SCID and BALB/c IL-4Rα^-/-^IL-5^-/-^, for long-term adult *B*. *malayi* parasite generation in comparison to the standard *M*. *unguiculatis* gerbil model. We utilised adult male and female *B*. *malayi* generated in the best performing rodent line to optimise a mammalian cell co-culture system for long-term maintenance over a time-course of 4 weeks. A primary human lymphatic endothelial cell co-culture with adult female *B*. *malayi* was selected and initially validated as a drug screening system against macrofilaricidal and *Wolbachia* depleting drugs.

## Materials and methods

### Ethics statement

All animal experiments were approved by the ethical committees of the University of Liverpool and Liverpool School of Tropical Medicine (LSTM) and conducted under Home Office Animals (Scientific Procedures) Act 1986 (UK) requirements. Animals had free access to food and water throughout the duration of studies, checked daily for welfare and weighed weekly.

### Animals

BALB/c IL-4Rα^-/-^IL-5^-/-^ mice were generated by crossing IL-4Rα^-/--^ and IL-5^-/-^ mice (Jackson Laboratories). Male CB.17 SCID mice were purchased from Charles River, UK. *Meriones unguiculatus* (Mongolian gerbils; jirds) breeding pairs were originally purchased from Charles River, Europe. Animal stocks were maintained under specific pathogen-free (SPF) conditions at the biomedical services unit (BSU), University of Liverpool, Liverpool, UK. Male BALB/c IL-4Rα^-/-^IL-5^-/-^ and CB.17 SCID mice were 6–8 weeks old and weighed 18–24 g at start of experiments. Male gerbils were 4–6 months old and weighed 80–100 g at start of experiments.

### *Brugia malayi* parasite maintenance

The life cycle of *B*. *malayi* (*Bm*) was maintained in *Aedes aegypti* mosquitoes and Mongolian gerbils. For *Bm* larvae (*Bm*L3), microfilariae (mf) were collected from gerbils via catheterisation. Gerbils were anaesthetised with isoflurane and subjected to peritoneal washes with RPMI 1640 medium (ThermoFisher Scientific) to harvest mf. Microfilariae were then purified using PD10 size exclusion columns (Amersham), enumerated by microscopy and mixed with human blood to a final concentration of 20,000 mf/ml. Microfilariae were fed to female *A*. *aegypti* mosquitoes through an artificial membrane feeder heated to 37°C (Hemotek). Blood fed mosquitoes were reared for 14 days with daily sugar-water feeding to allow development to *Bm*L3 stage. At day 14, *Bm*L3 were collected from infected mosquitoes by stunning at 4°C, then crushed and concentrated using a Baermann’s apparatus and RPMI medium.

### *B*. *malayi* experimental infections

For *Bm*L3 infection, male Mongolian gerbils, aged 4–6 months, were injected via the intraperitoneal route, with 400 highly motile *Bm*L3. Alternatively, male IL-4Rα^-/-^IL-5^-/-^ mice or male CB.17 SCID mice, both aged 6–8 weeks, were injected with 150 *Bm*L3. Animals were left for periods between 12- and 52-weeks post-infection to allow infections to proceed to the chronic adult stage.

### Adult *B*. *malayi* implantation surgeries

*Brugia malayi* adults were collected from infected donor CB.17 SCID mice via peritoneal lavage post-mortem. Parasites were then separated into male and female, washed with pre-heated, sterile RPMI (Sigma) and collected into groups of 10 female parasites for implantation (total n = 6–8). CB.17 SCID mice were then placed under surgical anaesthesia using isofluorane and received a subcutaneous injection of buprenorphine prior to implantation of female parasites into the peritoneal cavity. Implantation was achieved by making a small incision into the skin and abdominal cavity in the upper right quadrant and inserting parasites into the lower abdominal quadrant using a glass pipette to ensure all parasites were maintained in the cavity. The incisions were then re-sutured after implant and animals were re-housed as before and monitored closely.

### *In vitro* culture of *Brugia malayi* adults

For parasite cultivation, adult *B*. *malayi* were isolated from the peritoneal cavities of BALB/c IL-4Rα^-/-^ / IL-5^-/-^ mice via peritoneal lavage post-mortem. Parasites were washed with pre-warmed, sterile RPMI and separated into males and females, with worms of the highest motility and similar lengths selected for culture.

Initial culture studies evaluated the survival of female and male *B*. *malayi* on different endothelial cell monolayers; lymphatic human microvascular endothelial cells derived from human dermis (HMVECdly; LEC; Lonza), human embryonic kidney cells (HEK293; European Collection of Authenticated Cell Cultures; ECACC), and their recommended cellular media; endothelial basal medium plus supplements (EGM-2 MV; Lonza), and Dulbecco’s Modified Eagle Medium (DMEM; Sigma) for LECs and HEKs, respectively, to function as relative cell-free controls conditions in order to determine optimum culture conditions and longevity of culture systems. Individual cell lines were plated and allowed to form confluent monolayers in 6-well plates (Corning). At this point, parasites were added at a density of 2 adults per well with the addition of 6 ml of corresponding cell media containing 10% FBS, 2% penicillin-streptomycin and 2% amphotericin B. All cultured parasites were compared against parasites of the same age, freshly isolated from mice, to ensure cultured parasites were compared against an appropriate *in vivo* control. Culture media was replenished every 3 days and cells were replenished every week. Parasite motility was analysed every day using a 4-scale scoring system devised by Rao and Weil [21]. Parasites, both *in vitro* and *in vivo*, were taken for viability (MTT) and *Wolbachia* load analysis (qPCR) at the time points indicated in the corresponding results section.

### THP-1 macrophage co-culture

To determine whether addition of macrophages to the culture system enhanced parasite survival further, a monocyte-derived cell line, THP-1 (ECACC), originally derived from an acute monocytic leukaemia patient were evaluated. To differentiate THP1 cells into macrophages (Mϕ), cells were treated with 10 ng/ml of 12-O-tetradecanoylphorbol-I3-acetate (PMA; Peprotech), as previously described [22]. For polarisation into either a classically- or alternatively-activated phenotype, cells were treated with 50ng/ml recombinant interferon-gamma, (IFN-γ), or 25 ng/ml of recombinant interleukin 4 (IL-4) and interleukin 13 (IL-13), respectively (Peprotech), as previously detailed [23]. Forty-eight hours post-stimulation, Mϕ(IFN-γ), Mϕ(IL-4/IL-13), or non-polarised Mϕ(naïve) cells were washed 3 times in fresh medium and 0.4 μm pore-size transwell inserts (Corning) were added to fresh monolayers of LECs in a 6-well plate. Six millilitres of EGM-2 MV medium was then added, ready for the addition of parasites, as detailed above. Lymphatic endothelial cell monolayers with additional LEC trans-well inserts were included as controls.

### 3-(4, 5-dimethylthiazol-2-yl)-2,5-diphenyl tetrazolium bromide (MTT) viability assessments

To assess parasite viability quantitatively, parasites were removed from culture and washed in pre-warmed phosphate buffered saline (PBS; Gibco). Individual parasites were then transferred into separate wells of a 96-well plate and incubated with 0.5 mg/ml MTT (Sigma) for 2 hours at 37°C. Following incubation, parasites were washed in PBS, and incubated for 1 hour with 100% Dimethyl Sulfoxide (DMSO; Sigma) to solubilise the blue formazan product. The plate was then analysed using a fluorescent plate reader set at 450nm, including a primary shake step. Data were expressed as a percentage change in optical density readings from the *in vivo* control median.

### qPCR for *Wolbachia* quantification

DNA extractions were performed on individual parasites using a Qiagen QIAmp DNA mini kit in accordance with the manufacturer’s instructions. Quantification of *Bm* wsp copy numbers was performed using qPCR as previously described [15].

### Anti-*Wolbachia* drug screen validation

To assess the *in vitro* culture system’s ability to evaluate anti-*Wolbachia* drug activity, a drug challenge was carried out using the ‘gold-standard’ reference drug, doxycycline (DOX; Sigma Aldrich). Flubentylosin (ABBV-4083, also referred to as ‘tylosin A microfilaricide’; ‘TylAMac’ [24, 25]), a novel oral tylosin macrolide derivative compound proven to have superior *in vitro* and *in vivo* anti-*Wolbachia* activities compared with DOX, was synthesised as described [25] and assessed in comparison to DOX. Doxycycline was solubilised in ddH_2_O and prepared to a concentration of 5 μM in EGM-2 MV medium, whilst the same concentration of flubentylosin was solubilised in 100% DMSO and diluted in EGM-2 MV medium, equating to a final percentage of 0.05% DMSO per well. Drugs were replenished at each medium change. An equivalent percentage of DMSO used in the drug groups was added to vehicle control groups. Parasites were scored daily for motility and survival, with treatment end points at 7 and 14 days. An additional wash-out group, which entailed drug treatment for 7 days followed by a 7-day washout period was included in the study to evaluate any *Wolbachia* recrudescence. Quantitative PCR analysis, as previous, was employed at endpoints to evaluate *Wolbachia* reductions in response to treatment.

### Direct-acting macrofilaricide drug screen validation

To evaluate the system’s functionality in determining direct acting macrofilaricide activity, Flubendazole (FBZ) and Suramin (SUR) (both Merck) were used as reference drugs. Compounds were prepared at 10 μM in DMSO. The equivalent DMSO concentration was added to control wells. Parasites were maintained for 14 days in culture with daily motility scoring and an end-point MTT readout.

### Statistics

Data were tested for normal distribution using D’Agostino & Pearson omnibus normality tests. Data that passed normality tests were analyzed by one-way ANOVA with Tukey’s multiple comparisons tests. Data significantly different from a normal distribution were analyzed using Kruskal-Wallis with Dunn’s multiple comparisons tests. Significance was defined at alpha <0.05 and analyzed using GraphPad Prism v7.0h. Survival curves were compared using Mantel-Cox log-rank tests.

## Results

Severe-combined and IL-4Rα^-/-^IL-5^-/-^ immunodeficient mice are superior animal models for the production and long-term maintenance of adult *B*. *malayi* compared with *M*. *unguiculatus*.

**Fig 1:**
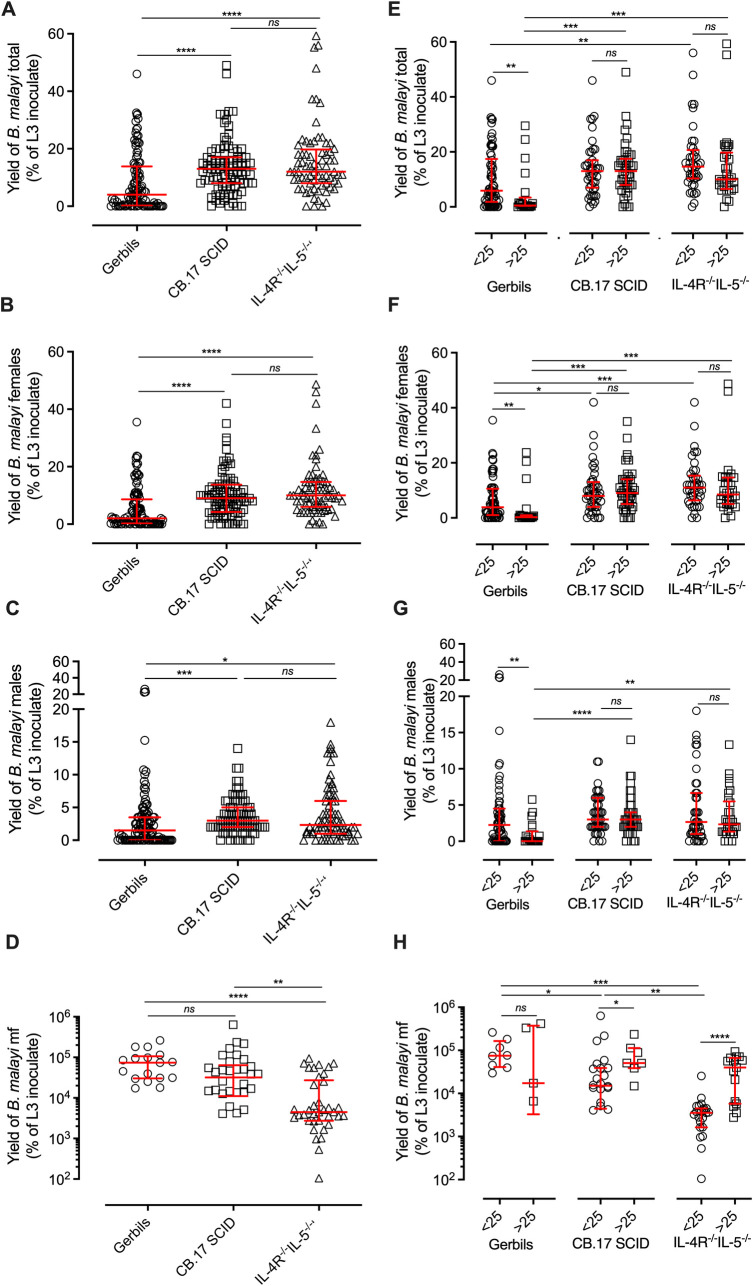
Meta-analysis of filarial adult parasitological yields in gerbils, CB.17 SCID or BALB/c IL-4Rα^-/-^IL-5^-/-^ mice. (A) Total, (B) female or (C) male *B*. *malayi* recovered from the peritonea of gerbils, CB.17 SCID or BALB/c IL-4Rα^-/-^IL-5^-/-^ mice. Data are number of worms as a % of initial larval inoculation. (D) Total peritoneal mf yields recovered from gerbils, CB.17 SCID or BALB/c IL-4Rα^-/-^IL-5^-/-^ mice adjusted per female *B*. *malayi* recovered. (E-H) data stratified into infections of <25 & >25 weeks. Each point represents an individual animal, red horizontal lines represent median and inter-quartile range values. Significance is indicated as ****P<0.0001, ***P<0.001, **P<0.01, *P<0.05 (Kruskal-Wallis with Dunn’s multiple comparison’s tests comparing between rodent models or Mann-whitney tests comparing between time points).

Parasitological data from gerbil, CB.17 SCID and BALB/c IL-4Rα^-/-^/IL-5^-/-^ mouse *B*. *malayi* intra-peritoneal infections were collated from 32 independent experiments (Fig 1 and Table 1). Length of experimental infections ranged between 12–52 weeks post-infection. Active infection was observed in 80.8% of gerbils (n = 105), 95.5% of CB.17 SCID mice (n = 88), and 95.9% of BALB/c IL-4Rα^-/-^IL-5^-/-^ mice (n = 72). The percentage of infections producing males was 65.4% in gerbils, 92.1% in CB.17 SCID and 87.5% in BALB/c IL-4Rα^-/-^IL-5^-/-^ mice. The sex ratio of infection was skewed towards females across all three groups, with a 1:0.65 ratio in gerbils, 1:0.34 in CB.17 SCID mice and 1:0.35 in BALB/c IL-4Rα^-/-^IL-5^-/-^ mice (Table 1). Adult worm recoveries were expressed as a percentage of the initial *Bm*L3 inoculation, to normalise for variable inoculations of *Bm*L3 between experiments. Both CB.17 SCID and BALB/c IL-4Rα^-/-^IL-5^-/-^ mice yielded significant, 3-fold higher adult worm burdens compared with yields recovered from gerbils (gerbil median recovery of inoculate = 4%, CB.17 SCID = 13%,BALB/c IL-4Rα^-/-^/IL-5^-/-^ = 12% (*P*<0.0001 Kruskal-Wallis with Dunn’s post-hoc test, Fig 1A). Yields of female *B*. *malayi* in CB.17 SCID and BALB/c IL-4Rα^-/-^IL-5^-/-^ mice were 4-5-fold higher compared with gerbils; gerbil median recovery of inoculate = 2% (median of absolute numbers of female parasites recovered = 4, range = 0–85), CB.17 SCID = 9% (median of absolute numbers of female parasites recovered = 8, range = 0–22); BALB/c IL-4Rα^-/-^/IL-5^-/-^ = 10% (median of absolute numbers of female parasites recovered = 10, range = 0–73) (*P*<0.0001 Kruskal-Wallis with Dunn’s post-hoc test, Fig 1B). Male *B*. *malayi* yields were 2- and 1.3-fold significantly higher in CB.17 SCID (median = 3%, median of absolute numbers of male parasites recovered = 2, range = 0–14) and BALB/c IL-4Rα^-/-^/IL-5^-/-^ mice (median = 2.3%, median of absolute numbers of male parasites recovered = 3, range = 0–20), respectively, in comparison to gerbils (median = 1.5%, median of absolute numbers of male parasites recovered = 7, range = 0–105) (P<0.001 Kruskal-Wallis with Dunn’s post-hoc test, Fig 1C).

**Table 1:** Summary of *B*. *malayi* adult parasitology in gerbils, CB.17 SCID mice and BALB/c IL-4Rα^-/-^IL-5^-/-^ mice.

Strain	% active infection (n)	Median % adult recovery of L3 inoculate (range, n)	% infections producing males (n)	Average ratio F:M
**Gerbil**	80.8 (105)	4% (0–32.5, 105)	65.4 (105)	1:0.65
**CB.17 SCID**	95.5 (88)	13% (0–49, 88)	92.1 (88)	1:0.35
**IL4Rα** ^-/-^ **IL-5** ^-/-^	95.9 (72)	12% (0–59.3, 72)	87.5 (72)	1:0.34

Adult parasitology summary between rodent strains and species detailing: 1. the percentage of active infections as a function of the total number of animals of that species/strain infected (the percentage of animals with parasites retrieved at necropsy), 2. the median percentage of adults recovered (males and females) as a function of the initial L3 inoculate, 3. the percentage of total infections producing males as opposed to female only infections, and the average ratio of females to males across infections.

*B*. *malayi* mf production in the peritoneum was normalised to number of female *B*. *malayi* worms recovered per animal, to allow for accurate comparisons. Significantly higher mf productions were observed in gerbils, which were 5-fold and 20-fold higher than those of CB.17 SCID or BALB/c IL-4Rα^-/-^/IL-5^-/-^ mice; gerbil median = 7.4x10^4^ mf/female (median absolute mf yields /animal = 856,666, range = 0–5,250,000) *vs* = 1.5x10^4^ mf/female (median absolute mf yields /animal = 238,700, range = 0–938,925), or 3.5 x10^3^ mf/female, (median absolute mf yields /animal = 61,000, range = 0–520,000), CB.17 SCID or BALB/c IL-4Rα^-/-^/IL-5^-/-^ respectively, P<0.0001, Kruskal-Wallis with Dunn’s post-hoc test, Fig 1D). Despite varying mf recoveries across species and strain, all mf displayed a normal, fully motile phenotype post-mortem, indicating no differences in health.

Parasitological data was stratified into different age-of-infection time-points (12–25 weeks and >25 weeks) to scrutinise whether differences between yields in gerbils and immunodeficient mice varied with chronicity of patent infection (Fig 1E–1H). At periods between 12–25 weeks post-infection, the total percentage of *B*. *malayi* adults recovered from both immunodeficient mouse strains were significantly (>2-fold) higher than those recovered from gerbils ((CB.17 SCID median = 13% (median of absolute number of parasites recovered = 9, range = 0–29), BALB/c IL-4Rα^-/-^/IL-5^-/-^ median = 13% (median of absolute number of parasites recovered = 12, range = 0–32) *vs* gerbil median = 5.9% (median of absolute number of parasites recovered = 21, range = 0–130), P<0.002 Kruskal-Wallis with Dunn’s post-hoc test, Fig 1E)). Female yields were significantly higher in both immunodeficient mouse strains compared with gerbils ((CB.17 SCID median = 8% (median of absolute number of females recovered = 6, range = 0–22), BALB/c IL-4Rα^-/-^/IL-5^-/-^ median = 11% (median of absolute number of females recovered = 9, range = 0–26) *vs* gerbils median = 3.9% (median of absolute number of females recovered = 6, range = 0–84), P<0.0001 Kruskal-Wallis with Dunn’s post-hoc test Fig 1F)), whereas there were no significant differences in male recoveries (BALB/c IL-4Rα^-/-^/IL-5^-/-^ median of absolute number of males recovered = 2, range = 0–11, CB.17 median of absolute number of males recovered = 2, range = 0–11, gerbil median of absolute number of males recovered = 21, range = 0–130, equating to median percentage recoveries of 15%, 13% and 6%, respectively) (Fig 1G)). In infections >25 weeks, the percentage of *B*. *malayi* adults recovered were significantly increased (20-fold) in both mouse strains compared to gerbils ((CB.17 SCID median = 13%; median of absolute numbers of parasites recovered = 13, range = 0–49) BALB/c IL-4Rα^-/-^/IL-5^-/-^ median = 10% (median of absolute number of parasites recovered = 14.5, range = 0–89) *vs* gerbils median = 0.5% (median of absolute number of parasites recovered = 13.5, range = 0–118), P<0.0001 Kruskal-Wallis with Dunn’s post-hoc test Fig 1E)). The number of females yielded were 28-fold lower in gerbils than from the two mouse strains ((CB.17 SCID median = 9%, median of absolute number of female parasites recovered = 9, range = 0–35) and BALB/c IL-4Rα^-/-^/IL-5^-/-^ median = 8.5% (median of absolute number of females parasites recovered = 11.5, range = 0–73) *vs* gerbils median = 0.3% (median absolute number of female parasites recovered = 6, range = 0–95) P<0.0001 Kruskal-Wallis with Dunn’s post-hoc test, Fig 1F)). Male yields were also significantly lower (3-fold) in gerbils than both mouse strains ((CB.17 SCID median = 3% (median absolute number of males recovered = 3, range = 0–14), BALB/c IL-4Rα^-/-^/IL-5^-/-^ median = 2.3% (median absolute number of males recovered = 3, range = 0–14) *vs* gerbils median = 0.1% (median absolute number of males recovered = 7, range = 0–40), P<0.0001 Kruskal-Wallis with Dunn’s post-hoc test Fig 1G)). In comparison, mf production in 12–25 week infected gerbils were 5- and 16-fold higher compared to CB.17 SCID and BALB/c IL-4Rα^-/-^/IL-5^-/-^ mice *vs* CB.17 SCID mice, ((gerbil median = 7.4x10^4^ mf/female (median absolute mf yield/animal = 983,817, range = 230,303–525,000), CB.17 SCID = 1.5x10^4^ mf/female (median absolute mf yield/animal = 155,295, range = 0–872,000), and BALB/c IL-4Rα^-/-^/IL-5^-/-^ = 4.5x10^3^ mf/female (median absolute mf yield/animal = 35,637.5, range = 0–228,228) P<0.0001 Kruskal-Wallis with Dunn’s post-hoc test Fig 1H)). In older, >25-week infections, no significant differences in mf production per female worm were apparent between the different models (gerbil median absolute mf yield/animal = 850,000, range = 0–4,306,000; CB.17 SCID = 510,987, range = 61.3–938,925; BALB/c IL-4Rα^-/-^/IL-5^-/-^ = 223,000, range = 39,000–5,200,000).

### Human lymphatic endothelium monolayers extend life-span of *B*. *malayi* adults in culture

**Fig 2:**
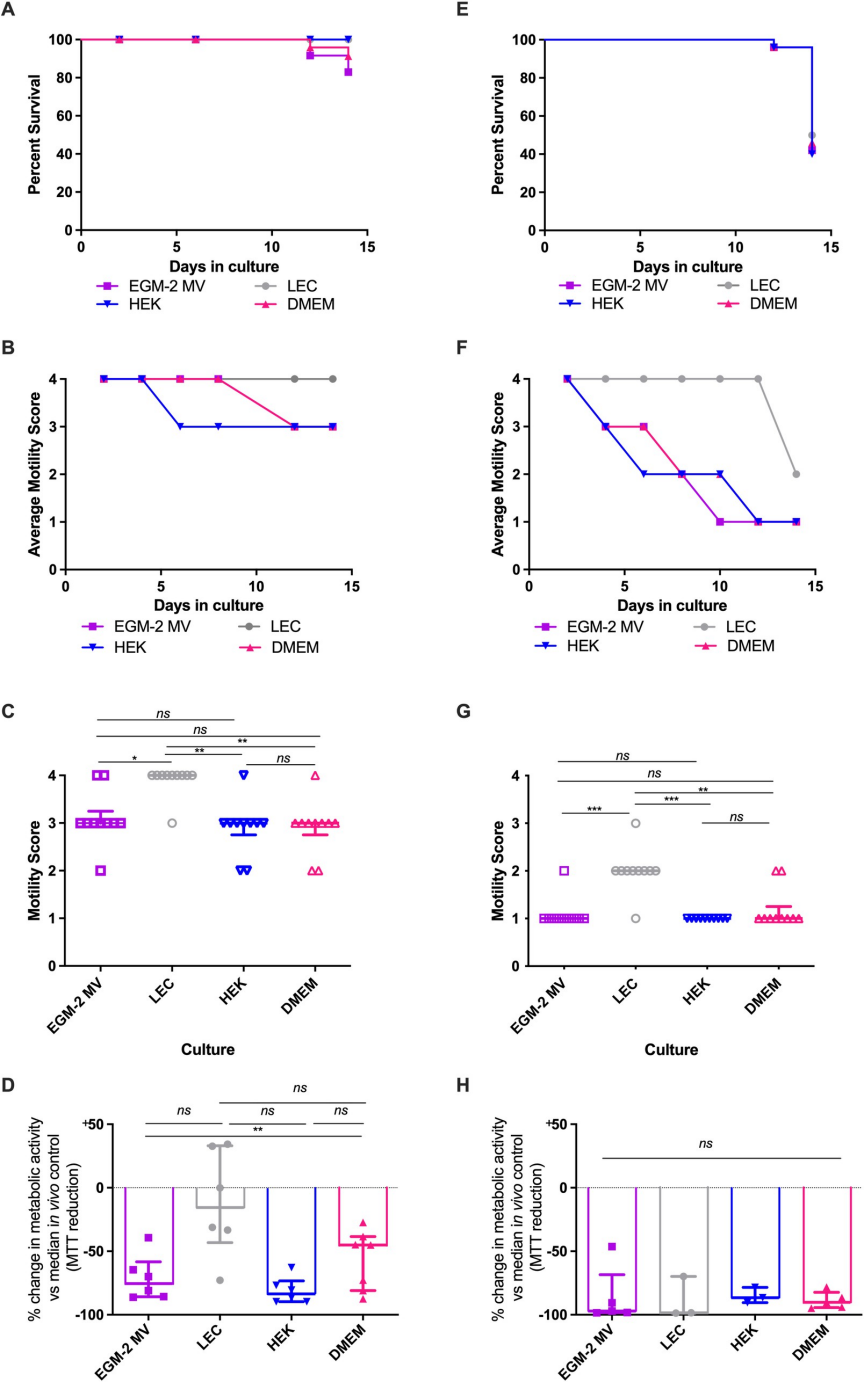
female and male *B*. *malayi* survival, motility and metabolic activity during culture with different cell and media conditions. (A) Kaplein-Meier survival or (B) average motility of female *B*. *malayi* over 14 days. (C) Individual female *B*. *malayi* motility scores or (D) MTT reductase activity of cultured female *B*. *malayi* at day 14, normalized to median MTT reductase activity in a sample five freshly isolated female *B*. *malayi* (median optical density = 0.48). (E) Kaplein-Meier survival or (F) average motility of male *B*. *malayi* over 14 days. (G) Individual male *B*. *malayi* motility scores or (H) MTT reductase activity of cultured male *B*. *malayi* at day 14, normalized to median MTT reductase activity in a sample five freshly isolated male *B*. *malayi* (median optical density = 0.32). Data is combined from 24 culture wells initially containing 48 viable filariae (A,E) or between 6–12 culture wells initially containing 12–24 viable filariae (B-D and F-H). Horizontal bars represent the median with interquartile range. Significance is indicated ***P<0.001, **P<0.01, *P<0.05 (Kruskal-Wallis with Dunn’s multiple comparison’s tests).

Initial experimentation was conducted to determine lifespans of male and female *B*. *malayi* adult parasites in culture following isolations from IL-4Rα^-/-^IL-5^-/-^ mice. Mammalian cell monolayers of human embryonic kidney cells (HEK-293) or human adult dermal lymphatic microvascular endothelial cells (referred to as lymphatic endothelial cells; LEC) were used to test whether their inclusion extended adult *B*. *malayi* survival, motility and metabolic activity to levels comparable to typically recorded within adult worms of the same age freshly isolated from rodent models. Cell co-cultures were compared with corresponding cell growth media only (Dulbecco’s Modified Eagles Medium + 10% foetal calf serum; DMEM+10%FCS or endothelial growth medium 2 microvascular, EGM-2MV, Fig 2). By day 14, females cultured on LEC monolayers displayed high rates of survival (46/48 surviving, 96%, Fig 2A). Other culture conditions showed lower survival by day 14 (HEK = 38/48, 79%, DMEM = 36/48, 75%, EGM-2MV = 32/48, 67%) although survival was not significantly different between culture conditions (Fig 2A). Average (median) motility of surviving female *B*. *malayi* worms in culture declined from day 6 (HEK) or day 12 (DMEM and EGM-2MV) whereas average motility of females cultured on LECs reflected the vigorous motile phenotype typical of freshly isolated worms (Fig 2B). When evaluated at day 14, average motility was significantly higher compared with other culture conditions (Kruskal-Wallis with Dunn’s comparison post-hoc test, P = 0.0006, Fig 2C). By comparing the metabolic activity of cultured *B*. *malayi* females against those freshly recovered from mice, female worms cultured on LEC monolayers displayed, on average, a non-significant 15% reduction in MTT reductase activity (Fig 2D). In comparison, the metabolic activity of female *B*. *malayi* cultures under other cell-culture conditions exhibited a 58–83% reduction in metabolic activity. The decline in metabolic activity in female *B*. *malayi* cultured on HEK cells was significant compared to female *B*. *malayi* cultured on LECs (P = 0.0098, Kruskal-Wallis with Dunn’s comparisons test Fig 2D).

Male *B*. *malayi* deteriorated more rapidly than female *B*. *malayi* in culture. At day 14, survival was 50% (24/48 males) on LEC monolayers, 40% (19/48) on HEK monolayers, 46% (22/48) in DMEM and 42% (20/48) in EGM-2MV (Fig 2E). Surviving male *B*. *malayi* cultured on LEC monolayers displayed an average fully motile phenotype until day 12 (Fig 2F) whereas motility immediately began to decline in other culture conditions. At day 14, average motility, whilst sub-optimal, was significantly higher in *B*. *malayi* male worms cultured on LECs than all other culture conditions (Kruskal-Wallis statistic = 38.48, P<0.0001, with Dunn’s multiple comparisons test, Fig 2G). *Brugia malayi* males in all culture conditions had drastically reduced MTT reductase activity in comparison to freshly isolated *in vivo* controls (adult worms) at day 14 (Fig 2H).

### A transwell co-culture system further improves female *B*. *malayi* survival in culture

**Fig 3:**
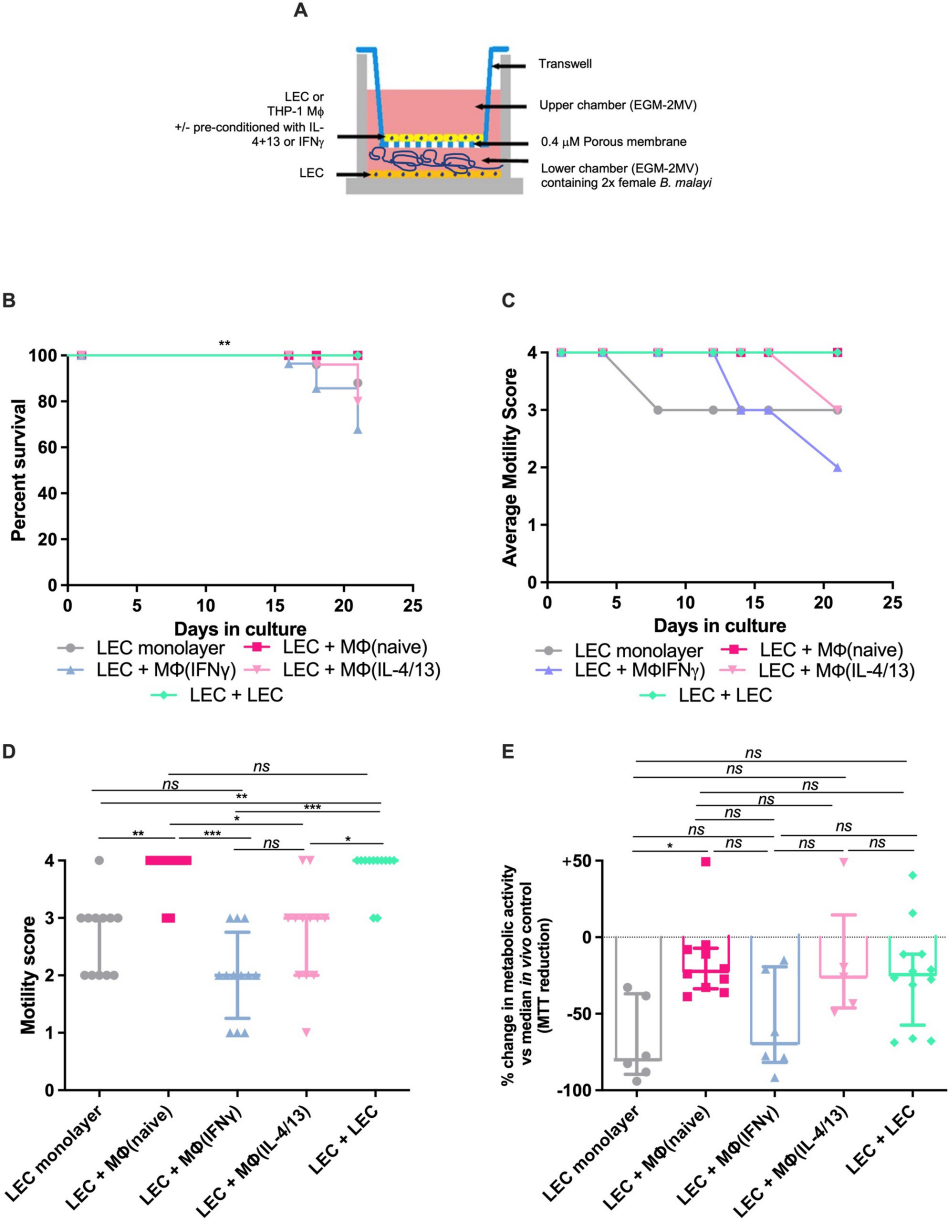
21 day trans-well adult *B*. *malayi* female co-culture system with macrophage leukocytes or LEC bilayers. (A) schematic of 2D trans-well co-culture system (B) Kaplein-Meyer survival curves or (C) average motility scores over 21-day culture period. (D) individual motility scores or (E) MTT reductase activity at day 21 normalized to median MTT reductase activity in a sample of five freshly isolated female *B*. *malayi* (median optical density = 0.48). Data is combined from 6 culture wells initially containing 12 viable female *B*. *malayi*. Horizontal bars represent the median with interquartile range. Significance is indicated ***P<0.001, **P<0.01, *P<0.05 (Mantel-Cox log-rank test or Kruskal-Wallis with Dunn’s multiple comparison’s tests).

In attempts to extend culture longevity of fully viable *B*. *malayi* females for periods ≥14 days, a lymphatic / leukocyte co-culture trans-well system was tested. These used a LEC monolayer in EGM-MV, as deemed the best performing culture (Fig 2), with the addition of a LEC or THP-1 human monocyte-derived macrophage (Mϕ) monolayer 6-well 0.4 μm pore transwell insert. THP-1 Mϕ were either seeded in a non-polarised state or conditioned with recombinant (r)IFN-γ or rIL-4/rIL-13, termed Mϕ(naïve), Mϕ(IFN-γ) or Mϕ(IL-4/13) (Fig 3A). Control LEC cultures (LEC monolayers) supported 100% survival (12/12 female *B*. *malayi*) for >16 days in culture (Fig 3B). Beyond this point, survival decreased slightly, with 92% (11/12) female *B*. *malayi* surviving by the 21-day end-point. Similar survival was apparent with LEC+Mϕ(IFN-γ) or LEC+Mϕ(IL-4/13) inserts, whereby 100% survival was maintained for 15 and 16 days (12/12 female *B*. *malayi*), before declining to 67% (8/12) and 83% (10/12), respectively, by day 21. Full survival was achieved for the duration of the 21-day culture period in LEC+Mϕ(naïve) or LEC+LEC insert co-cultures, a significant improvement on the other culture conditions tested (P = 0.0027 Mantel-Cox log-rank test). Full motility was sustained for the 21-day duration with the LEC+Mϕ(naïve) and LEC+LEC insert groups, whilst other groups demonstrated significant declines in motility during the culture period (P<0.0001, Kruskal-Wallis with Dunn’s comparison test Fig 3C and 3D). Analysis of metabolic activity in cultured worms at the day 21 end-point, compared against *in vivo* recovered female *B*. *malayi*, determined an 80% median reduction in metabolic activity of *B*. *malayi* cultured on LEC monolayers and a 69% reduction in female worms cultured in the presence of LEC+Mϕ(IFNγ) insert (Fig 3E). Metabolic activity was maintained to a level more representative of *in vivo* isolated worms in LEC+Mϕ(naïve), LEC+Mϕ(IL-4/13) and LEC+LEC insert co-cultures after 21-day incubations (22%, 26% and 24% reductions in metabolic activity, respectively). The improvement in metabolic activity supported by LEC+Mϕ(naïve) inserts was significant compared with LEC monolayers (P = 0.0132, Kruskal-Wallis with Dunn’s comparisons post-hoc test).

### Microfilariae production and *Wolbachia* titres in female *B*. *malayi* post-culture in LEC bilayers

**Fig 4:**
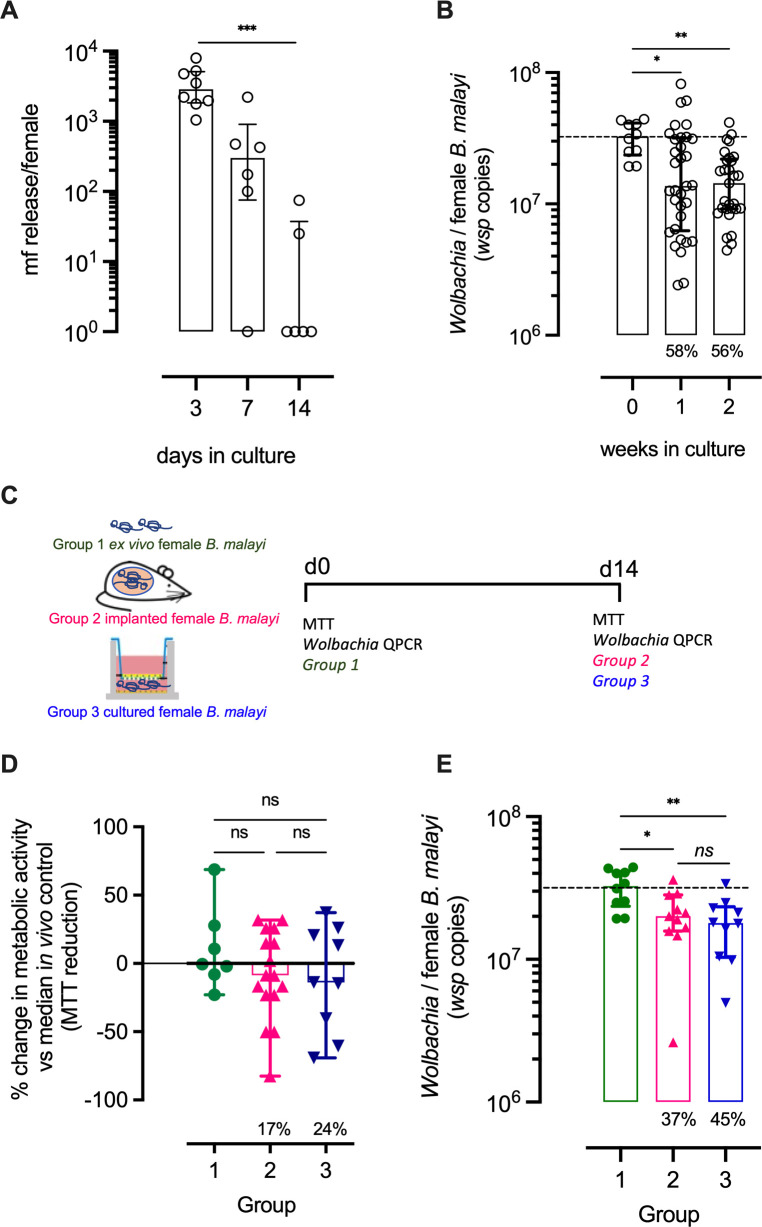
Comparison of *Wolbachia* titres over culture period in relation to *in vivo* recovered parasites. (A) Microfilariae release / female *B*. *malayi* at days 3, 7 and 14 after culturing in LEC bilayers (B) *Wolbachia* titres / *B*. *malayi* female removed from IL-4Rα^-/-^IL-5^-/-^ mice or (week 0) or maintained in LEC bilayer co-cultures for 1 or 2 weeks (C) schematic of experimental groups: group 1; female *B*. *malayi* freshly isolated from *in vivo* infection (CB.17 SCID mice), group 2; female *B*. *malayi* 2-weeks after single sex implantation into CB.17 SCID mice, group 3; female *B*. *malayi* 2-weeks after culture in LCE bilayers. (D) MTT reductase activity and (E) *Wolbachia* titres / *B*. *malayi* female derived from groups 1–3. Horizontal bars represent medians with interquartile range. Data is derived from a single experiment of 6 cultures (A) combined of three experiments of 6 cultures (B) or a single experiment of 12 cultures (D,E). Statistical significance determined by Kruskal-Wallis one-way ANOVA with Dunn’s post-hoc tests are indicated ns; not significant, *P<0.05, **P<0.01 and ***P<0.001.

Microfilariae release by female *B*. *malayi* into the lower chamber medium of LEC bilayers were assessed after 3, 7 or 14 days of culture (Fig 4A), in which mf production declined with time in culture. At day 3, a median of 2850 mf had been released per female *B*. *malayi*. This reduced to 300 mf per female at day 7 and 0 mf per female by day 14 (P<0.0001, Kruskal Wallis test). *Wolbachia* yields within female *B*. *malayi* after 1, 2 or 3 weeks in LEC+LEC insert co-cultures were compared with those from parasites immediately retrieved from IL-4Rα^-/-^IL-5^-/-^ mice (Fig 4B). After one to two weeks in culture, *Wolbachia* loads were significantly lower, on average by 55%, than *in vivo* controls (both 1.4 *vs* 3.13x10^7^ median *wsp* copies per worm) and by three weeks, levels had declined by 74.4% (0.8 *vs* 3.12x10^7^ median *wsp* copies per worm (P<0.005, Kruskal-Wallis with Dunn’s comparisons post-hoc test).

### Reductions in metabolic activity and *Wolbachia* content in female *B*. *malayi* after 2-week cultures in LEC bilayers reflect cessation of mf production

A 21-day culture period with a 6-well 0.4 μm pore transwell, LEC co-culture ‘bilayer’ had been defined as sufficient to maintain female *B*.*malayi* survival at 100% with full motility. However, metabolic activity, whilst not significantly different from *in vivo* isolated females, was reduced on average by 25% after two weeks in culture and *Wolbachia* content had declined significantly. As mf production was not sustained beyond 7 days *in vitro* (Fig 4A), the cessation of embryogenesis may have impacted on measures of overall metabolic activity and *Wolbachia* content, compared with freshly isolated, gravid female worms. To more accurately interpret whether these measures reflected a real decline in viability or cessation of mf production, a sample of 62 adult female worms were isolated from CB.17 SCID mouse infections, and approximately equal batches were either processed immediately for metabolic activity and *Wolbachia* molecular assays, set up into culture within LEC bilayers, or implanted into naïve CB.17 SCID recipients (Fig 4C). After removal of male worms and 2-week *in vivo* implantation, female worm mf production had ceased. This was confirmed by overnight culture release assays. Although ex-implanted female worms were fully motile, their total metabolic activity had reduced by 17% when compared against freshly isolated female worms from mixed-sex infections (0.55 *vs* 0.66 median O.D. Fig 4D). Median *Wolbachia* titres within female *B*. *malayi* ex-implanted worms were 37% significantly lower than those recovered from *in vivo* mixed-sex controls (2.0 *vs* 3.3x10^7^; P<0.05, Kruskal-Wallis with Dunn’s comparisons post-hoc test, Fig 4E). Reductions in metabolic activity and *Wolbachia* production in female-only explants after two weeks were similar to levels in *B*. *malayi* cultured on LEC bilayers, which displayed an average 24% decline in metabolic activity (median 0.5 O.D.), and a 45% reduction in *Wolbachia* content (1.8x10^7^ *vs* 3.3x10^7^). Levels of metabolic activity and *Wolbachia* were adjudged not to vary significantly between female ex-implanted worms and cultured parasites.

### Validation of *B*. *malayi* female LEC bilayer co-cultures to screen anti-*Wolbachia* drugs

**Fig 5:**
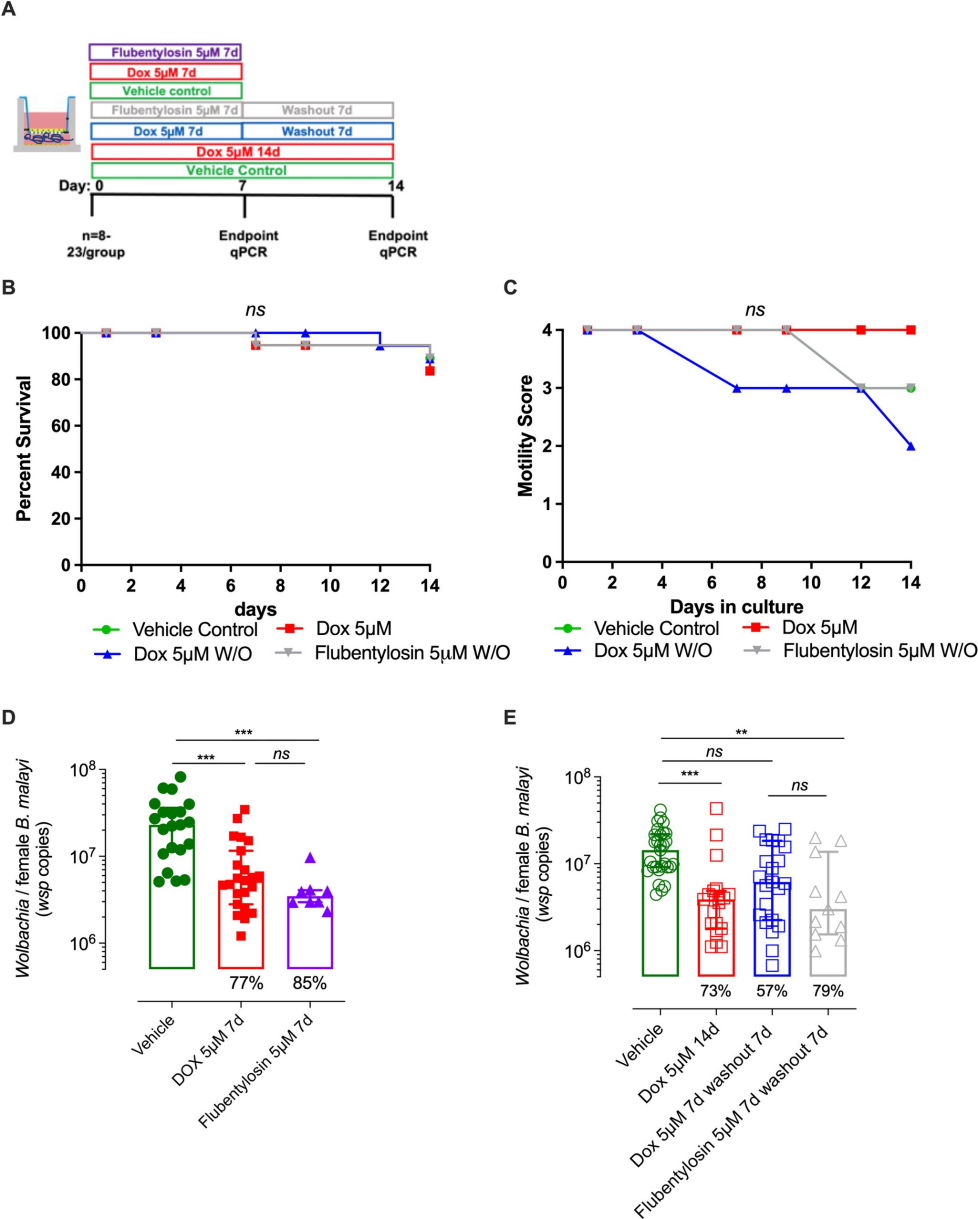
Validation of *B*. *malayi* female culture system as an anti-*Wolbachia* drug screen. (A) Schematic of anti-*Wolbachia* drug screen (B) Kaplein-Meier survival or (C) average motility of female *B*. *malayi* over 14 days to indicated exposures of doxycycline (DOX), TylAMac or vehicle (1% DMSO), (D) Anti-*Wolbachia* efficacy following 7 days or (E) 14 days of indicated exposures to DOX, TylAMac or vehicle. Data plotted is *wsp* copy numbers/*Bm* female determined by qPCR (D-E). Data is combined from two experiments with 6 culture wells initially containing 12 viable female *B*. *malayi* (for vehicle & DOX groups) or a single experiment of 6 culture wells for Flubentylosin groups. Horizontal bars represent median with interquartile range. Significance is indicated ***P<0.001, **P<0.01, *P<0.05 (Kruskal-Wallis with Dunn’s multiple comparison’s tests).

Doxycycline (DOX), and the anti-*Wolbachia* clinical candidate, flubentylosin (ABBV-4083), a novel tylosin analogue, were used to validate LEC bilayers as a screening system to evaluate anti-*Wolbachia* drug efficacies. Female *B*. *malayi* in LEC bilayers were dosed with drug at 5 μM for either 7-days or 14-days before evaluation of efficacy. In certain cultures, drugs were removed following 7-day exposure, parasites washed, and cultures extended for a further 7-day period. Due to significant decline in *Wolbachia* content with time in culture due to loss of mf production (Fig 4), vehicle treated cultures were ran simultaneously and sampled at 7 or 14 days as matched controls (Fig 5A). Negligible changes in survival were observed throughout the study duration (Fig 5A). Whilst there was some decline in average motility between days 7–14 in certain treatment groups, this was also observed in vehicle treated cultures, thus motility was not significantly influenced by DOX or flubentylosin drug exposure (Fig 5B and 5C). After 7-day treatment, both treatments significantly reduced *Wolbachia* levels. Doxycycline depleted *Wolbachia* on average by 77%, whilst treatment with flubentylosin reduced levels by 85% median depletion (5.26x10^6^ and 3.47x10^6^ median *Wolbachia* load per female *vs* 2.3x10^7^ vehicle control, P<0.0001, Kruskal-Wallis with Dunn’s comparison post-hoc test, Fig 5D). A further 7 days of DOX treatment maintained a significant 73% decrease in *Wolbachia* load (3.89x10^6^ *vs* 1.44x10^7^ median levels, P<0.0001, Kruskal-Wallis with Dunn’s comparison post-hoc test, Fig 5D). Removal of DOX at day 7 followed by a 7-day washout initiated a recrudescence of *Wolbachia* within the cultured female *B*. *malayi* worms, resulting in a 57% non-significant reduction of *Wolbachia* (Fig 5E). Contrastingly, 7-days post-removal of flubentylosin, *Wolbachia* recrudescence was not apparent, with a significant median *Wolbachia* reduction of 79% in comparison to the vehicle control group (3.02x10^6^ *vs* 1.44x10^7^ median *Wolbachia* loads, P<0.001, Kruskal-Wallis with Dunn’s comparison post-hoc test, Fig 5E).

### Validation of *B*. *malayi* female LEC bilayer co-cultures to screen macrofilaricidal drugs

**Fig 6:**
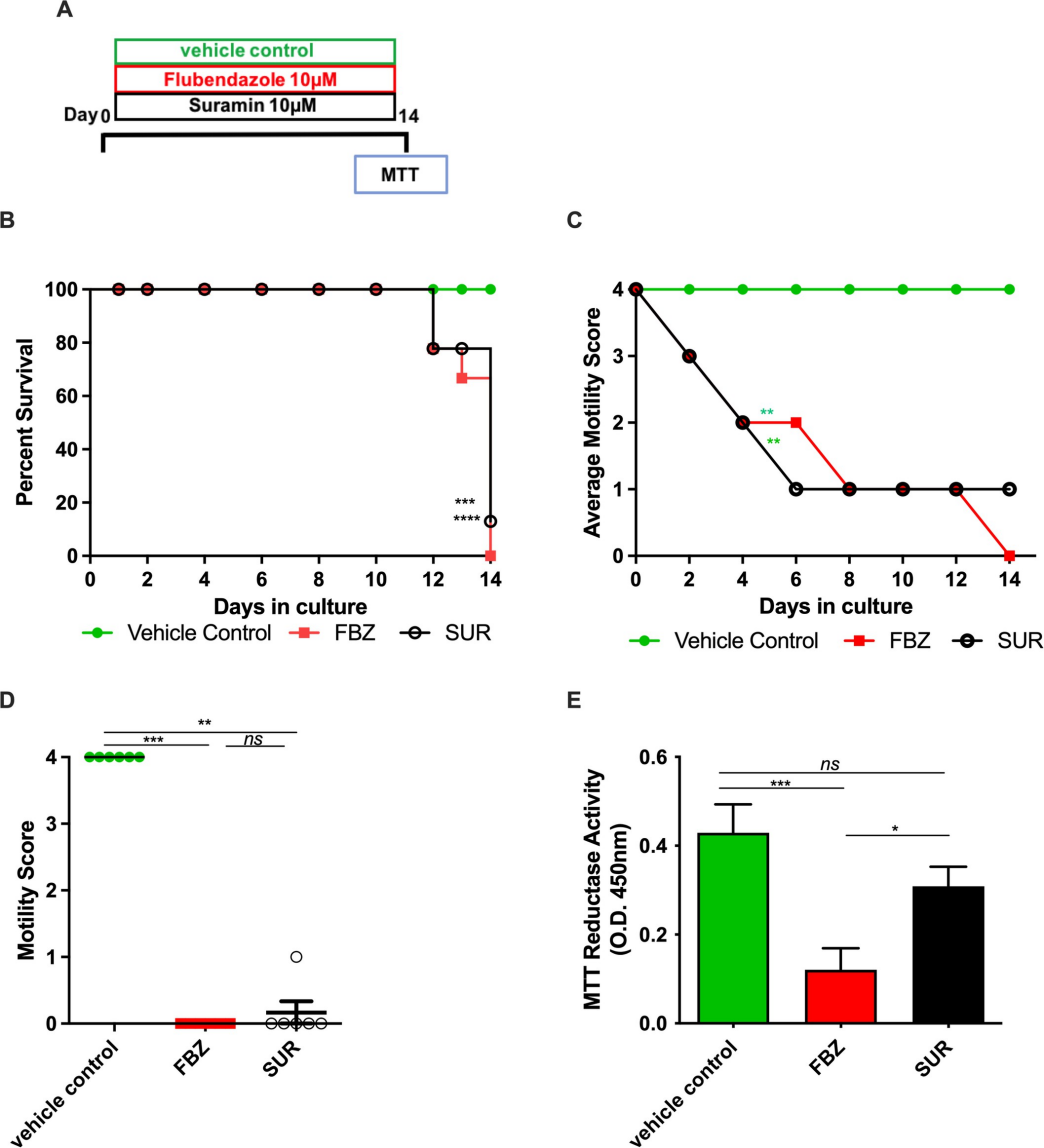
Validation of culture model as a direct-acting macrofilaricide drug model. (A) Schematic of macrofilaricidal drug screen (B) Kaplein-Meyer survival or (C) average motility scores of female *B*. *malayi* over 14 days during exposure to 10μM of flubendazole (FBZ), suramin (SUR) or vehicle (1% DMSO), (D) individual motility scores or (E) MTT reductase activity following 14 days of 10μM FBZ, SUR or 1% DMSO vehicle. Data is combined from two experiments with 6 culture wells initially containing 12 viable female *B*. *malayi*. Horizontal bars represent median with interquartile range. Significance is indicated ***P<0.001, **P<0.01, *P<0.05 (Kruskal-Wallis with Dunn’s multiple comparison’s tests.

Flubendazole (FBZ) and suramin (SUR), at fixed doses of 10 μM, were used to assess the functionality of the LEC bilayer co-culture system in evaluating direct macrofilaricidal activity (Fig 6A). Vehicle control treated female *B*. *malayi* retained 100% survival and motility throughout the course of the study, whereas survival declined from 12 days of treatment in both FBZ and SUR treatment groups with 12% surviving after 14 days of SUR treatment and 0% survival with FBZ treatment (P = 0.0019 & 0.0003, respectively, Log-rank Mantel Cox test; Fig 6B). Motility significantly decreased in both drug treatment groups, reducing to average scores of 0 or 1 by day 14 for FBZ and SUR, respectively (P = 0.0006, Kruskal-Wallis with Dunn’s comparison post-hoc test, Fig 6C and 6D). Quantitative metabolic activity assessment readouts at day 14 revealed significantly lower metabolic activity (72%) in FBZ-treated worms, which also had significantly lower activity in comparison to SUR treated worms, where 28% non-significant reduction compared to the vehicle control was observed (P<0.0001, Kruskal-Wallis with Dunn’s comparison post-hoc test, Fig 6E).

## Discussion

The discovery and development of new macrofilaricidal drugs for the prioritised filarial nematode NTDs: *B*. *malayi*, *B*. *timori*, *O*. *volvulus* and *W*. *bancrofti*, is hampered by a lack of long-term *in vitro* culture models to robustly test compounds for their effectiveness against the target adult life-cycle stage. This is reflected by deficits in culturing systems to reliably support the full development of human adult filariae from insect-derived, third-stage infective larvae *in vitro*, and a paucity of permissive laboratory models to propagate large quantities of male and female adult worms *in vivo*. ‘Two-dimensional’ (2D) cultures with human serum and/or monolayers of various mammalian cell lines are sufficient to support the initial growth of larvae through the third and potentially fourth cuticle moults for *B*. *malayi* [26], *O*. *volvulus* [27] or *W*. *bancrofti* [28]. In these studies, whilst certain adult internal organ morphological features can be microscopically determined, growth is severely retarded, with length typically ≥20-fold stunted compared to typical *in vivo* adult parasites. Moreover, sexual maturity and reproduction is not evidenced in these culture experiments. In one exceptional study, the technical feasibility of growing physiologically relevant, sexually mature adult *B*. *malayi* over 100 days, using cultures supplemented with a single-source of human serum has been demonstrated [29]. However, these data have not been reproduced using similar culture conditions, which is perhaps explained by variation in growth factors essential for filarial development in different sources of human serum [30]. More complex, 2D multi-cell, insert co-culture systems and 3D skin organoid culture scaffolds have been tested for *O*. *volvulus* which support ‘pre-adult’ stages surviving in culture for months, yet these *in vitro* generated filariae still display a severely stunted phenotype (typically <1mm) not dissimilar to growth achieved when culturing long-term on monkey kidney cell (LL-CMK2) monolayers [11, 31].

With no reliable way of propagating physiologically relevant filarial adult stages from larvae, the only viable approach available for drug screening *in vitro* is to isolate adult filarial worms from animals that are either naturally, or experimentally infected with filariasis. For onchocerciasis, several closely related cattle *Onchocerca* species can be utilised. The female intra-nodular niche is unsuitable for *ex vivo* culture and only by careful collagenase digestion of excised nodules can culture of female stages be supported long-term [32]. Due to their greater abundance within nodule tissues and their natural migratory behaviour between nodules, male *Onchocerca spp*. are more readily isolated and can survive for prolonged periods of several months, when cultured on LL-CMK2 monolayers [32]. This system, using the cattle parasite, *O*. *gutturosa*, has become adopted as the gold-standard *in vitro* screen to evaluate nematodicidal or anti-*Wolbachia* drug candidates [33–36]. However, limitations of the screen are throughput via sourcing infected cattle, mainly within Sub-Saharan Africa, and differential macrofilaricidal susceptibilities between *Onchocerca* and lymphatic filariae (as determined for drugs such as moxidectin and albendazole) [37–40]. Incidence of differential macrofilaricidal activities between male and female worms is another caveat to reliance on this screening system alone [41]. Excised *B*. *malayi* derived from experimentally infected *Meriones unguiculatus* (Mongolian gerbils) exhibit rapid loss of viability in serum-supplemented media which is reflected by upregulation of a number of stress-response genes over the first five days in culture [42]. A recent study utilising image-based motion analysis demonstrated decline in female *B*. *malayi* motility (a late-stage indicator of *in vitro* fitness) begins at 5–7 days in optimised serum supplemented medium culture [43]. Due to this rapid demise *in vitro*, moderate automated throughput screening utilising motility phenotype as a readout of *Brugia* female macrofilaricidal activity has been limited to a three-day exposure window [44]. This limits hit-compound identification to more rapid-acting agents with drug compounds exerting more gradual killing effects being over-looked. Further, titrations to determine minimum effective concentrations of hit-compounds may generate under-estimates of true efficacy following longer-exposures or washout time-frames. Finally, compounds synergising with artefactual *Brugia* physiology induced by sub-optimal culturing may risk driving identification of false-positive hit compounds.

For these reasons, in this study we wished to advance a more robust and long-term culture model of lymphatic filariasis for drug screening applications. Our aims were to 1) improve *in vivo* propagation by examining whether yields of adult *B*. *malayi* could be increased with inbred immunodeficient mouse strains, 2) assess lymphatic endothelium / leukocyte 2D co-culture systems to improve the longevity of adult worms in culture, and 3) evaluate reference macrofilaricidal drug responses in long-term culture.

Through an extensive meta-analysis of 32 independent sub-periodic human strain *B*. *malayi* infection experiments undertaken in our laboratory between 2012–2018, including untreated or vehicle control data derived from published *in vivo* preclinical model development, drug screening or immunological investigations [6, 15, 18–20, 23, 25, 45–49], we determined that immunodeficient mouse strains serve as improved models for propagations of adults. Whilst we have previously described reduced variability of adult worm burden and less frequent occurrence of infection failures in CB.17 SCID mice compared with gerbils [18], we extend our conclusions here that CB.17 SCID mice afford a >3-fold more productive source of adult infections per unit inoculation. Improvements in yields become more pronounced with chronicity of infection with >10-fold increases in adult burdens in >25-week old infections. In addition, we determine that an additional selective immunodeficient mouse model (BALB/c IL-4Rα^-/-^/IL-5^-/-^) could persistently yield greater burdens of adult *B*. *malayi* compared with gerbils, similar to productivity in CB.17 SCID mice. This long-term susceptibility reflects the profound loss of eosinophil-specific mediated immunity in these mice, controlled by IL-4 receptor and IL-5, as detailed in our prior investigations [20]. Increased susceptibility to adult filarial infections in IL-4R^-/-^/IL-5^-/-^ mice has also been determined for the related filarial parasites, *L*. *loa* and *S*. *sigmodontis* [50, 51]. In both mouse models, we noted an increased bias toward female *B*. *malayi* infections which might indicate residual immune-mediated or other host-dependent factors influencing male survival in mice. We also noted that initial mf accruement within the peritoneum in the first few months of patency, normalised per adult female worm, was most abundant in gerbils, with CB.17 SCID mice being intermediate and IL-4R^-/-^/IL-5^-/-^ mice displaying significantly reduced mf burdens. Whilst this might appear paradoxical given the increased adult burdens in mice, raised mf burdens in gerbils may reflect more balanced sex ratios in this model. In addition, other adaptive immune-mechanisms distinct from the IL-4 receptor and IL-5 signalling axis and lacking in gerbils and CB.17 SCID mice may exert immune-pressure on mf survival in IL-4R^-/-^/IL-5^-/-^ mice. In contrast to developing larvae, *B*. *malayi* mf activate IFN-γ producing lymphocytes in stage-specific BALB/c mouse experimental infections [52]. Our data indicate that CB.17 SCID or BALB/c IL-4R^-/-^/IL-5^-/-^ mice offer superior alternatives to gerbils specifically where a large demand for mature female *B*. *malayi* in *ex vivo* research is required, such as drug screening. In our experience, the reduced costs to produce BALB/c IL-4R^-/-^/IL-5^-/-^ mice in-house and more prolonged infection courses without incurring animal welfare issues compared with CB.17 SCID mice make this research model most suitable to propagate adult female *B*. *malayi*.

From our initial culture studies, we determined that co-cultures with human primary LEC monolayers extended the period of full motility and survival of mature female *B*. *malayi* to 14 days. Further analysis of metabolic activity confirmed that female adult *B*. *malayi* displayed only minor reductions compared with freshly isolated parasites from BALB/c IL-4R^-/-^/IL-5^-/-^ mice. This extended, fully viable life-span in culture was specific to the presence of LEC cells in culture as neither full endothelial growth medium nor human embryonic kidney cells could emulate the extended survival time. Our data is consistent with a recent publication demonstrating benefit of co-culture with a mouse endothelial cell line monolayer in augmenting survival of mf and adult *L*. *sigmodontis* [8]. Because in this study conditioned medium derived from endothelial cell cultures supported full survival of adult worms for 15 days, a hypothesis was proposed that endothelial cell-restricted macromolecules and metabolites released into culture provide a necessary source of nutrition to support *in vitro* adult worm fitness [8]. An alternative hypothesis proffered is that host cell monolayers may modify oxygen content in the liquid phase which favours filarial nematode survival [30]. Whilst LEC monolayers also extended the male *B*. *malayi* life-span *in vitro*, males cultured declined in viability from 12 days, and metabolic activity, motility, and percentage survival was severely reduced by day 14. We conclude that, unlike *Onchocerca spp*., it is more challenging to extend male *vs* female lymphatic filarial life-spans *in vitro*. Whilst the reasons for this are not clear, we speculate the smaller size and rapid motility of male *B*. *malayi* may make them more vulnerable to depletion of inherent energy stores which they are unable to sustainably replenish in culture.

Upon selection of a favourable monolayer and *B*. *malayi* adult-stage, we wished to test whether a mixture of leukocytes and lymphatic endothelium could emulate the lymphatic / lymphoid parasitic niche of the lymphatic system to further prolong longevity in culture. We selected a monocytic-Mϕ human THP-1 cell line to test as representative of abundant circulating or patrolling monocytes within the circulatory / lymphatic systems as well as major resident populations within afferent lymphatic–lymph node junctions [53]. Thus, we hypothesised monocyte-macrophage lineage cells would naturally occur in intimate and frequent contact with lymphatic-dwelling filariae *in vivo*. Further, because lymphocytes are absent, yet peritoneal Mϕ constitute as much as 50% of the white blood cells found within the long-term permissive CB.17 SCID *B*. *malayi* infection model, we speculated that this leukocytic cell may provide a source of nutritional factors suitable for supporting long-term parasite survival in the absence of a strong eosinophil larvicidal/adulticidal immune effector response. Finally, in support of this hypothesis of the Mϕ as a ‘feeder cell’, in earlier studies, we observed improved survival of *B*. *malayi* larvae when cultured with peritoneal Mϕ [54]. Because LF patients display a spectrum of T cell activation states that can influence the activation and metabolic status of Mϕ via differential cytokine production [54–56], we artificially polarised THP-1 Mϕ toward a classical (M1) or alternative (M2) phenotype with pre-exposure of recombinant IFNγ or IL-4/IL-13 [23]. We determined that non-polarised Mϕ in an upper chamber with LEC mono-layers in contact with filariae in the lower chamber improved full female *B*. *malayi* survival and motility for 3-weeks and maintained metabolic activity at approximately 75% of *in vivo* levels, indicating that these cells may provide an additional source of soluble factors to prolong adult lymphatic filarial survival *ex vivo*. Mϕ conditioned with IL-4/13 also appeared somewhat beneficial. Contrastingly, IFNγ conditioned Mϕ were marginally detrimental in *B*. *malayi* female *in vitro* fitness at 3 weeks, compared with LEC monolayers. This perhaps reflects production of soluble released mediators by classically-activated Mϕ, such as nitric oxide, which are toxic to adult *B*. *malayi* in culture [57, 58]. Surprisingly, the inclusion of a further LEC mono-layer in the upper chamber re-capitulated full survival, motility and sustained metabolic activity observed with non-polarised Mϕ+LEC co-cultures.

Given the simplicity of working with a single versus a double cell system, we opted to take forward the LEC bi-layer system for further validation as a drug screening system, including for anti-*Wolbachia* compound assessments. However, the transwell co-culture system could be of benefit in future applications to study other immune and non-immune cell types. We determined that *Wolbachia* content significantly declined with time in culture. *Wolbachia* are found both in somatic tissues of the hypodermis and the female germline, where populations expand within all embryonic stages [59, 60]. Female embryogenesis ceases in the absence of male mating *in vitro* (as evidenced by loss of mf production by 14 days in culture). By implanting single sex female worms into CB.17 SCID mice for periods matching those in culture (and thus blocking fertilisation *in vivo*) before estimating total *Wolbachia* content, we deduced that *Wolbachia* decline reflected a natural loss of embryogenesis and mf production. Further, the minor impact in metabolic activity of cultured *B*. *malayi* within LEC bi-layer cultures were similar to reductions following cessation of the embryogenesis pathway *in vivo;* a biological pathway of high metabolic demand. Thus, we conclude that notwithstanding loss of mf gestation, our *in vitro* LEC bi-layer system supports fully viable female *B*. *malayi* in culture for three weeks.

We subsequently demonstrated the advantage of being able to sustain fully viable *B*. *malayi* females for prolonged periods *in vitro* to accurately discriminate efficacy of anti-filarial drugs using physiologically relevant fixed dose concentrations (5–10 μM). Over a two-week period, the system was able to discern the inferior efficacy of the slower-acting anti-*Wolbachia* agent, doxycycline, versus the more potent novel tylosin analogue, flubentylosin, over a one-week exposure interval, as previously determined in animal infection models [25, 45, 47, 61]. Following doxycycline drug removal at one week, *Wolbachia* levels recrudesced by two weeks, a phenomenon consistently observed when using sub-optimal dosing of tetracyclines or rifamycins to treat *Brugia* or *Litomosoides in vivo* [25, 45, 47, 61]. Observing *Wolbachia* re-expansion within female *B*. *malayi* following drug removal is confirmation of the *in vitro* health of the *Wolbachia*-filarial symbiosis in our culture system. Thus, we have confidence that this culture system could be applied in the future to evaluate anti-*Wolbachia* candidates with putatively faster kinetics of symbiont depletion compared with registered antibiotics and to investigate mode-of-action and target identification studies. LEC bi-layer maintenance of female *B*. *malayi* was also successfully validated as an effective model in determining the long-term impact of the direct-acting nematodicidal agents, flubendazole or suramin. The pharmacology of flubendazole has been recently better fully characterised, whereby sustained release of injectable formulations for several weeks are necessary to exert significant macrofilaricidal activities against *Brugia* and *Onchocerca* filarial parasites in preclinical infection models [48, 62]. Conversely, it has been challenging to emulate flubendazole sustained exposure and macrofilaricidal activity *in vitro* or with mixed *in vitro* / *in vivo B*. *malayi* systems due to limitations in culture period [63, 64]. Our findings demonstrate that periods of two weeks in culture are necessary to determine macrofilaricidal activity of this benzimidazole drug at physiologically relevant dose concentrations. Therefore, our culture screening system might be useful to elucidate the pharmacodynamics of other members of the benzimidazole class against *B*. *malayi*, including albendazole [39] and oxfendazole [33] in deployment or in clinical development as alternative treatment strategies for lymphatic filariasis and onchocerciasis, respectively.

In conclusion, we have established an improved method for both *in vivo* propagations and *in vitro* maintenance of female *B*. *malayi*. We have demonstrated the utility of this approach for improving accuracy of assessing efficacy of anti-*Wolbachia* and direct-acting nematodicidal agents. The culture system could be deployed to more accurately evaluate the potential translation of ‘hit’ compounds against lymphatic filariasis following chronic drug exposures and to compare efficacy against *Onchocerca spp*. We recognise that the complexity of a 2–3 week-long LEC bi-layer culture system in current large well format (6-well) is unlikely to be adopted as a screen with moderate-throughput capacity. Miniaturisation into 24–48 well plates may be possible but will require onward careful evaluation of female *B*. *malayi in vitro* fitness over multiple weeks. As an alternative, this culture system may be incorporated as a secondary confirmatory screen to robustly validate initial hits identified in moderate screening systems and aid rational selection of compounds for advancement into *in vivo* preclinical testing. In this way, the screen may filter out false hit compounds that are synergising with stress responses in sub-optimal *in vitro* systems or compounds with differential active against surrogate life-cycle stages such as filarial mf. Similarly, LEC bi-layers can be utilised to determine efficacies that manifest more gradually at physiologically achievable dose concentrations prior to selection for animal testing. As an example application, we have utilised *B*. *malayi* female LEC bi-layers as a secondary screen following hit-identification against mf in the discovery of dihydrobenzoxazepinone compounds with broad-spectrum anthelmintic activities [65]. Further opportunities for innovation include incorporation of this long-term co-culture model with human hepatic organoids via a microfluidics physiological system (‘organ-on-a-chip’) to more accurately model drug metabolism and in vivo pharmacokinetics of late-stage candidate anti-filarial drugs. As such, the novel methodologies described herein, if adopted, should reduce overall animal use, lower costs and improve decision making toward expediting new curative drugs for filarial NTDS.
